# Metabolomics Adaptation of Juvenile Pacific Abalone *Haliotis discus hannai* to Heat Stress

**DOI:** 10.1038/s41598-020-63122-4

**Published:** 2020-04-14

**Authors:** Fei Xu, Tingting Gao, Xiao Liu

**Affiliations:** 1 Key Laboratory of Experimental Marine Biology, Center for Mega-Science, Institute of Oceanology, Chinese Academy of Sciences, Qingdao, 266071 China; 2 Fishery College of Zhejiang Ocean University, Key Laboratory of Marine Fishery Equipment and Technology of Zhejiang, Zhoushan, 316022 China; 3 University of Chinese Academy of Sciences, Beijing, 10049 China

**Keywords:** Marine biology, Animal physiology

## Abstract

Temperature fluctuation is a key abiotic factor for the growth and survival of Pacific abalone *Haliotis discus hannai*, particularly during climate change. However, the physiological mechanism underlying the abalones’ response to heat stress remains unknown. We sought to understand the metabolic adaptation mechanism of Pacific abalone to heat stress for further analyzing its heat tolerance capacity. For two groups experienced different acclimate temperature (10 °C and 30 °C for 62 days), the Pacific abalone juveniles displayed significantly different survival rates under 31 °C acute heat treatment. A total of 1815 and 1314 differential metabolites were identified from the 10 °C and 30 °C acclimate groups respectively, by comparing mass spectrometry data of the samples before and after heat stimulation. Heat stress led to mitochondrial failure, resulting in incomplete oxidative metabolism of amino acids and fatty acids in the mitochondria, and massive accumulation of unstable metabolic intermediates in cells. The 10 °C acclimated group accumulated more harmful substances after heat stimulation, provoking further stress responses and pathophysiological processes. In comparison, the 30 °C acclimated group showed stronger regulation capacity to produce beneficial substances for metabolic homeostasis. The findings provided insight into the heat response of marine animals, especially concerning mitochondrial metabolism.

## Introduction

Global warming will trigger more extreme weather events that can potentially be deleterious to marine animals, especially the ones distributed in the intertidal and neritic zones^1,2^. Organisms inhabiting this range face higher water temperature elevations and more environmental fluctuations during the summer season. Aquaculture animals, which are subjected to fluctuations in natural conditions, are more vulnerable to these physical and chemical stimulations as they are usually stocked intensively at high density and rely on water influx from coastal areas^3^. Even a slight change in the climate may cause enormous economic losses in mariculture. The analysis of thermal stress on aquaculture animals has thus gained more research interests^4–7^.

Pacific abalone *Haliotis discus hannai*, a cold water gastropod, is the predominantly farmed abalone species in many Asian countries^8^. It is naturally distributed in cold water, implying that high temperatures would limit its southern expansion along the Asian coast. In recent years, the dominant aquaculture strategy in China is transferring juvenile abalones to warmer south China during the winter, thereby accelerating the animal’s growth rate. In the meantime, the traditional genetic selection based on growth traits may contribute to a decreased heat stress tolerance of the organism. Consequently, the massive mortality in summer months has become a challenge that has led to an enormous economic loss in abalone and the entire molluscs industry in recent years^9^. While some overwintering abalones were transferred back to the northern region during spring, some were cultured for a whole year in the southern region where the seawater temperature approaches the heat tolerance limit of the organism in the summer. Both genetic breeding and aquaculture technologies altered the distribution region of the Pacific abalone. However, little is known about the metabolic response of Pacific abalone to thermal challenges and their adaptation mechanism. Thus, having a robust and accurate understanding of the effects of temperature on abalone energetic metabolism is imperative and critical for predicting the future impacts of the rising ocean temperatures on abalone farming.

For marine ectothermic organisms, environmental temperature could strongly affect the rate of virtually all metabolic processes, which is mediated mainly through the metabolic rate^10,11^. The physiological responses to thermal stress initially concentrate upon redeploying pathways to increase energy production^12,13^ and oxidation resistance^14^. Many factors may be related to heat tolerance in these organisms, including the rate and range of temperature change, animal size, state of ontogeny^15^, and heat exposure history^16,17^. At the same time, hereditary factors also play essential roles in the adaptation to thermal stress. South African abalone has significant genetic diversification^18^ and should have been a result of adaptive to different water temperatures between west and east coast^19^. The biochemical reaction rates within abalone tissues increase with temperature rise, which leads to an increase in the energy need to maintain homeostasis. Consequently, a coordinated response of various metabolic pathways and oxidation of multiple energy substances is required^20^. For instance, under a high-temperature environment, adenosine triphosphate (ATP) production is elevated in abalones by enhancing oxygen consumption and activating the pathways for anaerobic metabolism^12^. In addition, the accessorial rate and extent of protein^11^ and lipids^21^ oxidation are also redeployed for ATP production, for a stable equilibrium between energy output and energy consumption.

However, oxidative stress occurs when the adjustment of substrate oxidation fails to fully compensate for the increased demand for metabolism at higher body temperature. The consequent accumulation of reactive oxygen species can directly result in the oxidative damage of macromolecules, including lipids, proteins and nucleic acids^22^. The side effects of oxidative damage on cells can in part be abolished by cellular antioxidant defence systems or by the expression of heat shock proteins. Whereas the former mechanism requires antioxidases and antioxidants to degrade free radicals to substances with lower toxicity, the latter mechanism entails the repair of misfolded polypeptide chains of damaged proteins or the elimination of irretrievably damaged proteins. Both of these mechanisms have been described in abalones^23–25^.

The hepatopancreas is an organ of the digestive tract of arthropods and molluscs. Mollusc hepatopancreas plays an important role in energy storage, metabolite transformation, enzyme synthesis, and antioxidant protection^26^, indicating its potential as the ideal target tissue for the study of the metabolism mechanism of heat stress on abalones. We thus investigated the abalone’s hepatopancreas with metabolomics based techniques. By comparing the results of the phenotypic and metabolic differences between animals acclimated under different seawater temperatures, we analyzed the heat tolerance and adaptation mechanisms of the juvenile Pacific abalone.

## Methods

### Ambient seawater temperature data collection

Seawater temperature data were collected for two typical abalone culture regions in northern and southern China. The data for the northern abalone culture region was generated from the Xiaomaidao station of the National Marine Science Data Center (http://www.nmdis.org.cn/). The data for the southern region was from Fuzhou Marine Fisheries Bureau (http://hyj.fuzhou.gov.cn/), which averaged the seawater temperature from three aquaculture sites around Fuzhou city (Huangqi, Tongxin bay, and Minjiang estuary). Monthly average sea surface temperature data of these two regions in the past three years were collected.

### Animals and acclimatization

Pacific abalone juveniles (shell length: 26.2 ± 2.3 mm, shell width: 16.5 ± 2.5 mm, total wet weight: 2.17 ± 0.56 g) used in this study were purchased from a commercial farm in Qingdao (Shandong, China), and were kept in culture tank at 20 ± 0.3 °C for two-week recovery. The animals were then divided into two groups for a 62-day acclimatization period under 10 °C and 30 °C seawater controlled by a digital temperature controller. The decrease rate of seawater temperature was 1 °C per day, while the increase rate was 1 °C per day till 25 °C and then 0.5 °C per day to 30 °C. During this time, the seawater was aerated, and the salinity was maintained at 31 ± 1. Excess feeding with brown alga *Laminaria japonica* was given daily. The remaining food and excrement were removed the following day and renewed with clean, sand-filtered seawater at the corresponding temperature. After the 62-day acclimatization, animals from each acclimated group were recovered to 20 °C at a rate of + (-) 2 °C per day, and were then maintained for one week to make the L group (10 °C acclimated) and H group (30 °C acclimated) respectively.

### Acute heat treatment and sampling

The heat tolerance of L and H groups of juvenile abalones were compared by metabolomics assay and survival measurement after exposure to 31 °C seawater. A total of 72 individuals from each group were randomly selected and transferred into 31 °C seawater immediately. After three hours treatment, 27 individuals were randomly sampled from each group to make LH group (31 °C treated L group) and HH group (31 °C treated H group) for metabolomics assay, while the remaining 45 individuals in each group were continually cultured till 48 h for measuring survivals. Hepatopancreas tissues were dissected from four groups of juvenile abalones (L, H, LH, and HH). Within each group, tissues from three abalones were mixed and ground into powder in liquid nitrogen for subsequent metabolite extraction and metabonomic-based analysis. In this way, nine biological replications were produced for each group.

### Metabolite extraction

50 mg of sample was taken and placed in an Eppendorf tube, followed by the addition of 1000 μl extraction solvent (V_methanol_:V_acetonitrile_:V_water_ = 2:2:1, containing internal standard 2 μg/ml). The mixture was homogenized in ball mill for 4 min at 45 Hz, then ultrasound treated for 5 min (incubated in ice water). After homogenization for three times, the mixture was incubated for one hour at −20 °C to precipitate proteins. Centrifugation was performed at 12000 rpm for 15 min at 4 °C. A total of 825 μl supernatant was carefully transferred into a fresh Eppendorf tube and dried in a vacuum concentrator without heating. 200 μl extraction solvent (V_acetonitrile_:V_water_ = 1:1) was then added for reconstitution, followed by vortex 30 s, sonicated for 10 min (4 °C water bath), and centrifuge for 15 min at 12000 rpm (4 °C). 75 μl supernatant was transferred into a fresh 2 ml LC/MS glass vial for UHPLC-QTOF-MS analysis, while another 20 μl was taken from each sample and pooled as QC samples. Six technical replicates were conducted to QC sample with 75 μL each for UHPLC-QTOF-MS analysis. Total ion chromatogram and correlation analysis of quality control (QC) samples are presented in Supplementary Figs. 1–6

### Liquid chromatography tandem mass spectrometry analysis

LC-MS/MS analyses were performed according to previous report^27^. In brief, the UHPLC system (1290, Agilent Technologies) with a UPLC BEH Amide column (1.7 μm 2.1*100 mm, Waters) coupled to TripleTOF 6600 (Q-TOF, AB Sciex) was used. The mobile phase consisted of 25 mM NH_4_OAc and 25 mM NH_4_OH in water (pH = 9.75) (A) and acetonitrile (B) with gradient elution as follows: 0 min, 95% B; 7 min, 65% B; 9 min, 40% B; 9.1 min, 95% B; 12 min, 95% B, with solvent delivery at a fixed rate of 0.5 mL/min. The sample injection volume was 3 μL. The triple time of flight (TOF) mass spectrometer was used due to its ability to acquire MS/MS spectra on an information-dependent basis (IDA) during an LC-MS experiment. In this mode, the acquisition software (Analyst TF 1.7, AB Sciex) continuously evaluates the full scan survey MS data as it is collected and triggers the acquisition of MS/MS spectra depending on preselected criteria. In each cycle, 12 precursor ions whose intensities were greater than 100 were chosen for fragmentation at collision energy (CE) of 30 V (15 MS/MS events with product ion accumulation time of 50 msec each). The electrospray ionization (ESI) source conditions were set as follows: Ion source gas 1 as 60 Psi, Ion source gas 2 as 60 Psi, Curtain gas as 35 Psi, source temperature 600 °C, Ion Spray Voltage Floating (ISVF) 5000 V or −4000 V in positive or negative ion mode, respectively.

### Data preprocessing and annotation

According to previous report^27^, MS raw data files were converted to the mzXML format using ProteoWizard^28^, and processed by R^29^ package XCMS (version 3.2)^30^. The preprocessing results generated a data matrix that consisted of the retention time, mass-to-charge ratio (m/z) values, and peak intensity. R package CAMERA^31^ was used for peak annotation after XCMS data processing. In-house MS2 database and KEGG^32^, PubChem^33^, ChEBI^34^ and HMDB^35^ were applied in metabolites identification. The identified metabolites’ function annotations were excerpted from the description in HMDB, PubChem and UniProt^36^.

### Identification of differential metabolites

Orthogonal projections to latent structure-discriminant analysis (OPLS-DA) was applied to the analysis of the metabolite content to obtain more reliable relevant differential metabolites (DEMs) information between experimental groups referring to the method of Thevenot’s report^37^. The parameters of the evaluation of the OPLS-DA model included R^2^X, R^2^Y, and Q^2^Y. The R^2^X and R^2^Y represented the accounted rate of the developed model to the X and Y parameter matrices, respectively, while Q^2^Y indicated the predictive capability of the model to Y. The model was considered more reliable when R^2^X, R^2^Y, and Q^2^Y were closer to 1, and was considered adequate when Q^2^Y > 0.5 and excellent when Q^2^Y > 0.9. The model quality was evaluated by cross validation. A VIP value was given to each metabolite by OPLS-DA model to express the degree of its contribution to the model construction. In other words, the VIP value demonstrates the closeness of the metabolite and heat shock treatment to a linear relationship. DEMs were screened by combining fold change (FC), *P* values of Student’s *t*-test and VIP value of each metabolite based on OPLS-DA. A set of substances from differential expression analysis was referred to the DEM set, named as “A*-*B”. Metabolites with concentration higher in sample B than in sample A were designated upregulated DEMs, while those with a lower level in sample B compared with sample A were downregulated DEMs.

## Results

### Ambient seawater temperature of different abalone culture regions

**Figure 1:**
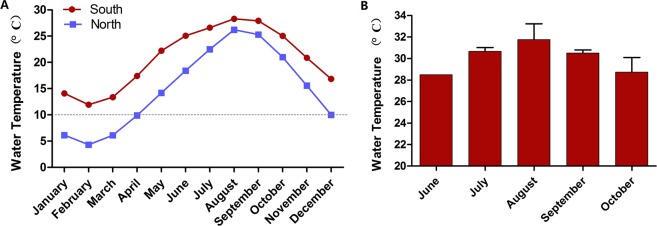
Seawater temperature data. (**A**) The monthly average temperature of south and north sites for the past three years. (**B**) The maximum temperature of the south site in summer months for the past three years.

Abalones cultured in the northern sea area experienced at least four months low temperature (averagely < 10 °C) annually (Fig. 1A), while that in the southern sea area experienced as many as five months hot temperature (average maximum seawater temperature was higher than 28 °C, Fig. 1B). The maximum water temperature in the Fuzhou aquaculture area was around 30 °C from July to September. In this context, acclimation temperatures were designed as 10 °C and 30 °C, to compare the heat response differences of abalones.

### Heat tolerance comparison

**Figure 2:**
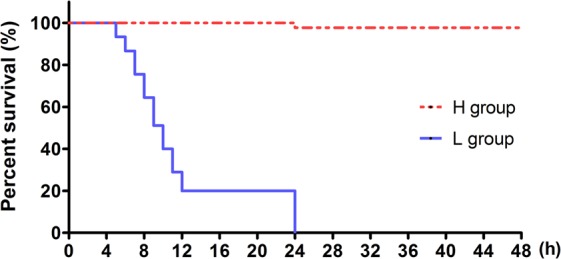
Kaplan-Meyer survival analysis of different Pacific abalone juveniles at 31 °C heat shock. L group, Pacific abalone juveniles acclimated for 62 days at 10 °C and then cultured for 7 days at 20 °C; H group, Pacific abalone juveniles acclimated for 62 days at 30 °C and then recovered 7 days at 20 °C.

Within the 48 hours of heat treatment at 31 °C, abalone juveniles from L and H groups displayed significantly different survival rates (Fig. 2). All animals from the L group died within 24 hours, showing high sensitivity to extremely high temperatures. Whereas, only one individual from the H group died within the 48 hours of observation, indicating tolerance to extremely high temperature.

### DEMs identification

**Figure 3:**
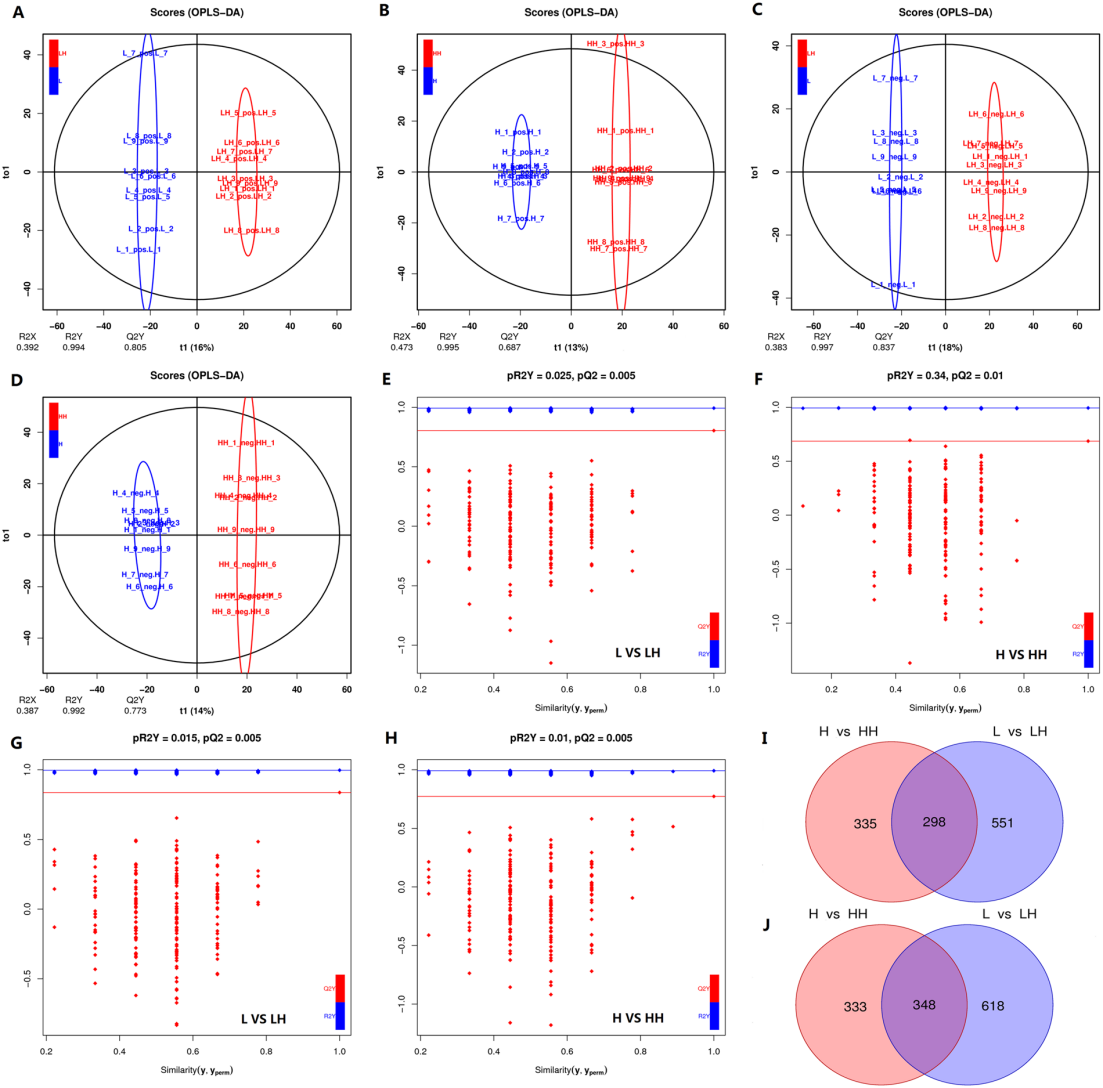
OPLS-DA model analysis and DEMs identification. (**A–D**) are OPLS-DA model analysis results (**A**,**B**) data from positive ion mode; (**C**,**D**), negative ion mode) based on LC/MS data of metabolites from Pacific abalone hepatopancreas samples of control (blue) and heat shock groups (red). Permutation test confirmed the validation of each model (**E–H**). (**I**,**J**) show Venn diagram analysis results of DEMs identified from different comparison pairs. L, Pacific abalone juveniles acclimated for 62 days at 10 °C and then cultured for 7 days at 20 °C; LH, Pacific abalone juveniles from L group treated 3 hours by 31 °C heat shock; H, Pacific abalone juveniles acclimated 62 days at 30 °C and then recovered after 7 days at 20 °C; HH, Pacific abalone juveniles from H group treated for 3 hours with 31 °C heat shock. Image was created by R software version 3.5.3 (https://www.r-project.org/).

OPLS-DA model distinguished group pairs well (L*-*LH and H*-*HH) with metabolites detected from either positive or negative ion mode (Fig. 3A–D, positive ion mode: R^2^Y_L*-* LH_ = 0.994, R^2^Y_H*-*HH_ = 0.995, Q^2^Y_L*-*LH_ = 0.805, Q^2^Y_H*-*HH_ = 0.687; and negative ion mode: R^2^Y_L*-*LH_ = 0.997, R^2^Y_H*-*HH_ = 0.992, Q^2^Y_L*-*LH_ = 0.837, Q^2^Y_H*-*HH_ = 0.773). Permutation test confirmed the validation (pQ^2^Y < 0.05) of OPLS-DA models (Fig. 3E–H). With *P* < 0.05 and VIP > 1 as threshold, 1815 (849 in positive ion mode and 966 in negative ion mode) and 1314 (633 and 681) DEMs were identified in L-LH and H-HH comparison pairs, respectively. Besides, there were 298 (positive ion mode) and 348 (negative ion mode) overlapped DEMs between the two comparison pairs (Fig. 3I,J).

### Metabolite biomarkers

**Figure 4:**
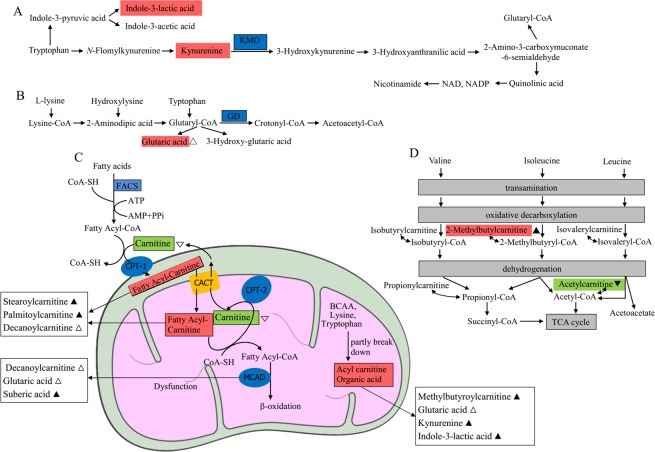
Effect of heat stress on metabolites in the mitochondria of juvenile Pacific abalones. ▲ indicates metabolites significantly increased in both L and H groups after heat stress; ▼indicates metabolites significantly decreased in both L and H groups after heat stress; △ and ▽ indicate metabolites significantly increased and decreased, respectively, in only L group after heat stress. FACS, fatty acyl-CoA synthase; CPT-1, 1-type carnitine palmitoyltransferase; CPT-2, 2-type carnitine palmitoyltransferase; CACT, carnitine acylcarnitine translocase; MCAD, medium chain acyl-CoA dehydrogenase; BCAA, branched-chain amino acid; GD, glutaryl-CoA dehydrogenase; KMO: kynurenine monooxygenase. Blue background indicates enzyme protein; orange background indicates channel protein; red background indicates significantly accumulated metabolites; green background indicates significantly reduced metabolites.

Within the 1815 and 1314 identified DEMs, annotated substances account for 247 (Supplementary Table S1) and 241 (Supplementary Table S2) in L-LH and H-HH comparison pairs, respectively. Stricter criteria were then used with FC > 1.5 or <0.6, *P* < 0.05 and VIP > 1.5 to identify metabolite biomarkers (MBs) within annotated DEMs. As a result, 42 substances (34 of which were upregulated and eight downregulated) were obtained in L-LH comparison pair (Supplementary Table S3), while 100 were selected in H*-*HH set (92 upregulated and eight downregulated, Supplementary Table S4). There were 28 MBs, which were all upregulated, overlapped by both comparison pairs (Table 1). Functional annotation on the 28 overlapped substances indicated the possible dysfunction of some metabolic enzymes either localized in the mitochondria or the cytoplasm. The mitochondrion enzymes possibly being influenced were mainly involved in the oxidative catabolism of amino acids and fatty acids (Fig. 4).

**Table 1:** Functional annotation of overlapped metabolite biomarkers between L*-*LH and H*-*HH sets.

No.	Name	Function	L*-*LH		H*-*HH	
			FC	VIP	FC	VIP
1	Nicotinamide	Component of the coenzyme NAD, cofactor, antioxidant, neuroprotective agent, anti-inflammatory agent.	11.53	2.08	11.21	1.55
2	(-)-Naringenin	Inhibitor to cytochrome P450 isoform CYP1A2.	8.74	1.68	6.22	1.77
3	O-Acetyl-L-serine	Takes part in cysteine and glutathione metabolism.	5.16	2.2	5.47	1.78
4	Maleamic acid	Conjugate acid of a maleamate.	5.94	2.08	5.06	1.74
5	2-Methylbutyroylcarnitine^*^	Intermediate of isoleucine metabolism.	9.55	2.36	4.55	2.25
6	Stearoylcarnitine^*^	Metabolite biomarker suggests carnitine palmitoyltransferase (CPT) II deficiency.	4.15	1.97	3.71	2.46
7	1,3-Dimethyluric acid	Metabolite from the metabolism of methylxanthines (caffeine, theophylline, and theobromine) mainly catalyzed by CYP1A2.	3.54	2.21	3.59	2.26
8	Iminodiacetic acid	Chelator of Ca^2+^, Mg^2+^ and polymer to create an ion-exchange resin.	3.6	2.34	3.45	2.38
9	Imidazoleacetic acid	Metabolite product of histamine metabolism.	1.52	1.7	3.45	2.38
10	1,7-Dimethyluric acid	A metabolite from the metabolism of methylxanthines (caffeine, theophylline, and theobromine) mainly catalyzed by CYP1A2.	2.19	1.78	3.33	1.6
11	CDP-choline	Essential molecule for membranes biosynthesis and repair, molecule for neuroprotection.	2.72	2.26	3.11	2.12
12	Amrinone	Acardiotonic, vasodilator.	2.08	2.04	2.8	2.06
13	DL-Indole-3-lactic acid^*^	Metabolite of tryptophan.	1.93	2.14	2.67	2.36
14	Suberic acid^*^	Metabolite biomarker suggests deficiencies of medium-chain acyl-CoA dehydrogenase, carnitine-acylcarnitine translocase, or malonyl-CoA decarboxylase.	1.85	1.76	2.34	2.14
15	alpha-Guanidinoglutaric acid^*^	Derivative of glutaric acid, which can induce seizures.	1.95	2.28	2.33	2.36
16	Creatine	Facilitator of ATP biosynthesis.	1.75	2.08	2.19	2.37
17	D-Biotin	An important component of enzymes involved in metabolizing fats and carbohydrates. Maintains cellular metabolism.	2.24	2.05	2.19	1.75
18	MDMA	Nervous stimulant. Facilitates ATP production.	1.96	2.16	2.11	2.24
19	Phenelzine	Non-selective and irreversible monoamine oxidase inhibitor, neuroregulator.	1.73	2.14	2.08	2.35
20	7-Methylxanthine	Metabolite from the metabolism of methylxanthines (caffeine, theophylline, and theobromine) mainly catalyzed by CYP1A2.	1.54	1.69	2.05	1.95
21	N-Acetyl-L-tyrosine^**#**^	Metabolite biomarker suggests aromatic L-amino acid decarboxylase deficiency.	1.92	1.72	2.04	1.59
22	Acamprosate	Metabolite could stabilize the chemical balance with side effect of irregular heartbeats.	2.11	1.85	2.03	1.6
23	Erythrono-1,4-lactone	Lactone of tetronic acid.	2.07	2.42	1.79	2.29
24	5-Methylcytidine	Post-transcriptional modifications found in tRNA, snRNA, and rRNA.	1.86	2.21	1.72	1.95
25	5-Hydroxyindoleacetate^**#**^	Metabolite involved in tryptophan metabolism. Breakdown product of serotonin.	1.98	1.75	1.72	1.84
26	Niflumic Acid	Inhibitor of chloride channels and T-type calcium channels. Inhibitor of phospholipase A2 and cyclooxygenase-2.	2.06	1.85	1.7	1.65
27	L-Kynurenine^*^	Intermediate metabolite of tryptophan. Metabolite biomarker suggests kynurenine-3-monooxygenase deficiency.	1.81	2.18	1.64	1.73
28	L-Palmitoylcarnitine^*^	Metabolite biomarker suggests carnitine palmitoyltransferase (CPT) II deficiency.	1.78	1.54	1.62	1.8

Note: HMDB, PubChem, and UniProt were applied in metabolites function annotation. ^*^Indicates metabolites associated with enzymes in mitochondria; ^**#**^Indicates metabolites associated with enzymes in cytoplasm.

Functional annotation of MBs specifically represented in each comparison pair was also conducted. In the L*-*LH set, the remaining six upregulated MBs after removing the 28 overlapped MBs were regarded as L*-*LH specific (Table 2). Similarly, 64 MBs were obtained as H-HH specific, in which the top 15 was listed in Table 2. There were significant differences regarding the functions of the two specific MBs sets, suggesting different heat response at metabolites accumulation level of L and H groups. The six MBs in the L*-*LH set suggested the occurrence of oxidative stress. In comparison, the top 15 MBs in the H*-*HH specific set consisted of substances beneficial to metabolic homeostasis, including various dipeptides, antioxidants, and neuroprotective substances.

**Table 2:** Functional annotation of set specific metabolite biomarkers.

Set	Name	Description	FC	*P*	VIP
L-LH	Cytosine	Pyrimidine base, product of CTP dephosphorylation. The CTP can transfer a phosphate to convert ADP to ATP.	5.71	8.37E-04	1.84
	L-Gulonic gamma-lactone	Direct precursor of vitamin C.	2.83	4.71E-04	1.85
	Phenoxybenzamine	Metabolite causes vasodilatation and increases cardiac output.	2.55	7.38E-03	1.68
	Lavandulol	Pheromone.	2.06	5.57E-04	2.00
	beta-Alanine	Neurotoxin, mitochondrial toxin, and metabotoxin when at a high level.	1.74	5.48E-05	2.09
	Glutaric acid	Product during metabolism of some amino acids. Act as an acidogen and a metabotoxin when at high levels.	1.62	1.66E-03	1.89
H-HH	Pro-Tyr	Dipeptide.	5.14	9.23E-04	2.15
	Pro-Asn	Dipeptide.	2.13	5.52E-04	2.15
	Thr-Ala	Dipeptide.	2.10	4.08E-04	2.09
	D-Glucose-6-phosphate	Metabolic hub.	3.20	1.37E-02	1.68
	Ammelide	Hydrolysis product of melamine.	2.33	7.10E-05	2.17
	S-Methyl-5’-thioadenosine	Metabolite influencing the regulation of gene expression, proliferation, differentiation, and apoptosis.	2.07	6.65E-03	1.83
	4-Hydroxycinnamic acid	Important source of antioxidants.	2.05	2.54E-05	2.31
	trans-2-Hydroxycinnamic acid	Important source of antioxidants.	2.02	5.25E-06	2.39
	Theobromine	Vasodilator, diuretic and heart stimulator.	2.01	6.87E-03	1.70
	N-Acetyl-D-glucosamine	Metabolite initiating a protective response to stress, modulating a cell’s capacity to grow and divide, and regulates gene transcription.	2.00	1.91E-03	1.90
	Tiopronin	Reducing agent, neuroprotective agent.	1.99	5.42E-05	2.23
	L-Phenylalanine	Metabolite could be converted to tyrosine, used in the biosynthesis of dopamine and norepinephrine neurotransmitters.	1.97	1.10E-02	1.66
	Dimethylglycine	Byproduct of the metabolism of choline.	1.95	6.11E-03	1.78
	Phe-Ala	Dipeptide.	1.92	1.74E-02	1.52
	Ajmalicine	Monoterpenoid indole alkaloid, vasodilator agent.	1.87	4.52E-03	1.8

Note: HMDB, PubChem, and UniProt were applied in metabolites function annotation.

The oxidative metabolism occurring in the mitochondria, the powerhouse of cells, is crucial to heat stress response and heat tolerance. Figure 4 shows the effect of heat stress on substances related to the metabolism of amino acids and fatty acids in the mitochondria of hepatopancreas tissues of juvenile abalones.

## Discussion

After experiencing two weeks of homogeneous environment culture, juvenile abalones from 30 °C acclimation group exhibited higher survival than 10 °C group, under short-term extreme heat stress (31 °C). The result highlights the role of acclimation in the heat stress response of abalones. We inferred that high-temperature acclimation exerts specific effects on metabolic processes in abalones, which allow them to respond quickly in a physiologically beneficial manner when exposed to heat stress^11^, predisposing them to survive in a harsh environment. Based on this hypothesis, a metabolomics study was conducted to further assay the physiological biochemistry adaptation mechanism of juvenile abalone to heat stress.

Homeostasis of physiological processes requires the activation of various metabolic pathways to increase energy supply for animals in response to environmental stress. Temperature changes can alter the biochemical reaction rate of many enzymatic processes in poikilothermic animals, whereas acute heat stress can induce prompt adjustment^38^. In this study, a variety of intermediates produced from incomplete degradation of amino and fatty acids were identified to be significantly accumulated in the hepatopancreas of thermally treated abalone juveniles (HH-group and LH-group), indicating that excessive ambient temperature leads to dysfunction of pathways utilizing protein and lipid oxidation as energy supply. This should exacerbate the energy shortage experienced by the heat-stressed animals. Mitochondria, the powerhouse of cells, is the main site for amino and fatty acids metabolism. Heat stress affects the normal function of mitochondrial oxidative metabolism^39^. It causes accumulation of intermediates, some of which were reported to disturb cell homeostasis and hinder cellular function, leading to pathophysiological processes such as muscle spasm, muscle weakness^40^, heart failure, cardiac arrest, and sudden death^41,42^.

As reported in previous studies, abalones experienced elevated ammonia excretion rate and weight loss when reared under high temperature^11,24^, suggesting the increased contribution of protein/amino acid oxidation to their energy supply. The metabolomics-based studies on small abalone *H. diversicolor*^43^, sea cucumber *Apostichopus japonicus*has^38,44^ also showed the influence of heat stress on amino acid metabolism. In this study, tissue concentrations of two tryptophan metabolic intermediates significantly increased after heat stress (Table 1): L-kynurenine and indole-3-lactic acid (3-IAA). In humans, the aromatic amino acid L-tryptophan has two main metabolic pathways (Fig. 4A). The kynurenine pathway accounts for more than 95% of tryptophan oxidative catabolism, while the other pathway includes a series of indole compounds^45^. The dysfunction or absence of the mitochondrial enzyme kynurenine-3-monooxygenase (EC: 1.14.13.9) results in a significant accumulation of kynurenine^46^, which is also associated with human neuromuscular dysfunction^47^. Kynurenine elevation in heat-treated abalone may reflect the breakdown of mitochondrial function and possible disorder of normal physiological function of the neuromuscular system. The data regarding the cytotoxicity of 3-IAA are limited. Some studies indicated that this metabolite could activate p38, c-Jun N-terminal kinases, caspase-8, caspase-9, and caspase-3 to induce cell apoptosis^48^. Another amino acid-related metabolic biomarker, glutaric acid, was identified to be increased in the LH group. The massive accumulation of glutaric acid is attributable to the dysfunction of many mitochondrial enzymes, including glutaryl-CoA dehydrogenase (EC: 1.3.8.6), electron transfer flavoprotein (ETF), and ETF-ubiquitin oxidoreductase (ETF-QO) (EC: 1.5.5.1)^49^. Glutaryl-CoA dehydrogenase catalyzes glutaryl-CoA to generate crotonyl-CoA and CO_2_ during the mitochondrial oxidative metabolism of lysine, tryptophan, and hydrolysine (Fig. 4B). Due to the dysfunction of this enzyme, glutaryl-Co A is directed toward glutaric acid production^50^. Excessive accumulation of glutaric acids and derivatives can cause a variety of neurometabolic and muscular disturbances^40^, partially explaining the poor performance of L group under heat stress.

Massive accumulation of substances related to fatty acid metabolism was also observed in this study. Two carnitine linked metabolites were identified in MBs: stearoylcarnitine and L-palmitoylcarnitine (Table 1), accumulation of which has been recognized to indicate the malfunction or absence of mitochondrial enzyme carnitine palmitoyltransferase II (CPT-2, EC: 2.3.2.21)^51^. Carnitine plays an obligate role in long-chain fatty acids transferring from the cytoplasm to the mitochondrial matrix for subsequent β-oxidation^52^. The import was mainly conducted by combined reactions of fatty acyl-CoA synthase (FACS, EC: 6.2.1.3), carnitine palmitoyltransferase (CPT-1 and CPT-2) and carnitine acylcarnitine translocase (CACT) (Fig. 4C)^52^. CPT-1 (EC: 2.3.1.21) is localized in the outer mitochondrial membrane, where it is responsible for exchanging the CoA for carnitine to produce fatty acyl-carnitines (e.g. stearoylcarnitine and L-palmitoylcarnitine) which then enters the mitochondrial matrix via facilitated diffusion by CACT. The fatty acyl-carnitine complex is then disrupted by CPT-2, and the fatty acid rebinds to CoA in the mitochondria, where the replaced carnitine then diffuses back across the membrane by CACT into the mitochondrial intermembrane space^53^. Consequently, CPT-2 dysfunction causes accumulation of long-chain fatty acyl-carnitines (e.g. stearoylcarnitine and L-palmitoylcarnitine) in cells while reducing the carnitine content^51^. Significantly diminished L-carnitine was indeed observed in the L-LH pair (Supplementary Table S1).

Furthermore, carnitine is also involved in the catabolism of branched-chain amino acid (e.g. isoleucine and valine)^52^. High level of 2-Methylbutyroylcarnitine (Table 1, Fig. 4D) is indicative of the function loss of the enzyme short/branched chain specific acyl-CoA dehydrogenase (EC: 1.3.8.-), which is responsible for catalyzing the third metabolic step in the pathway for mitochondrial oxidative catabolism of isoleucine and valine^54^. Elevated stearoylcarnitine, L-Palmitoylcarnitine, and 2-Methylbutyroylcarnitine content in the hepatopancreas of heated abalone juveniles indicated the accumulation of these intermediates in mitochondrial, instead of proceeding to downstream oxidation. Accumulation of long-chain fatty acyl-carnitines was also observed during cell apoptosis process^55^. Accumulated palmitoylcarnitines was reported to be able to activate caspase 3, 7, and 8, and initiate cell apoptosis^55^. Furthermore, fatty acids combined with carnitines can also affect redox chemistry, impair tissue function, and cause myocardial injury through perturbed membrane molecular dynamics, potentially triggering cardiac arrhythmia and cardiac arrest^41,42^.

The speculation of fatty acid oxidation disorders of abalone under heat stress was further supported by the observation of the suberic acid accumulation (Table 1)^56^ and significant accumulation of an intermediate of medium-chain fatty acid, Decanoyl-L-carnitine, in LH group (Supplementary Table S1). In humans, syndrome with hypoglycemia, hypoketonemia, cardiomyopathy, neuronal migration abnormalities, and other tissues pathological signature are usually presented during fatty acid oxidation disorders^57^. Hypoketonemia of abalone under heat stress was also observed in this study: significantly reduced concentration of acetoacetic acid in both L-LH and H-HH comparison pairs (Supplementary TableS S1 and S2). We failed to detect the other two main ketone bodies possibly because the volatile acetone is undetectable by the LC-MS/MS technology used in this study, while hydroxybutyric acid usually occurs at low concentration in animal cells. At the same time, neuronal migration abnormalities of heat-treated abalones, i.e. pathophysiological symptoms of rotating and shaking the shell, stiff muscles, and loss of adhesion, fainting, and cardiac arrest, were also observed in this study, consistent with observations in other studies and other *Haliotis* species^58^.

Besides the accumulation of more intermediates related to mitochondrial dysfunction in the LH group, LH and HH groups showed significantly different metabolic responses to heat stress. The LH group specifically accumulated some MBs harmful to cell homeostasis. Among the six L-LH specific MBs, L-Gulonic gamma-lactone is the substrate of the last step of the biosynthesis of vitamin C^59^, a powerful reducing agent. Its accumulation should indicate the deficiency of vitamin C biosynthesis ability under heat stress, if the abalone has any. Beta-Alanine is a neurotoxin, mitochondrial toxin, and metabotoxin at elevated levels in human nerve tissues^60^. However, H-HH specific MBs enriched some substances beneficial to metabolic homeostasis. S-Methyl-5’-thioadenosine is a metabolite that can influence the regulation of gene expression, proliferation, differentiation, and apoptosis^61^. Both 4-Hydroxycinnamic acid and trans-2-hydroxycinnamic acid were important sources of antioxidants^62,63^. Both theobromine and ajmalicine were regarded as vasodilators. N-Acetyl-D-glucosamine is a metabolite that can initiate a protective response to stress, modulate the cells’ capacity to grow and divide, and regulate gene transcription^64^. Tiopronin is a reducing agent and a neuroprotective agent. Juvenile abalones in the HH group showed quicker response to regulate the production of metabolic promotion and stabilization, cell injury repair agents, and neuroprotection after heat treatment, which could explain their adaptation mechanism to heat tolerance. However, more molecular studies on mitochondrial activities during heat response are still needed to support our findings.

The mitochondria function as the cellular powerhouse and are responsible for accommodating increased energy demand during heat stress. Therefore, the structure and function of mitochondria in the Pacific abalone need to be stabilized to sustain their tolerance to heat stress. The single population is limited to reflect the general heat response scenario of the Pacific abalone. Environmental metabolomics response in abalones is worthy of further investigations, as well as molecular studies on mitochondrial activities during heat response in marine animals.

## Conclusion

We studied the metabolic mechanism of heat tolerance in juvenile Pacific abalones. While comparing different groups with different acclimation history, we found that substances involved in the oxidation decomposition of amino acids and fatty acids were accumulated, which indicated dysfunction of mitochondrial enzymes under heat stress. Abnormal accumulation of these metabolites aggravates stress response and leads to pathophysiological processes, especially when the organisms face increased energy demand under conditions of increased ambient temperature. Individuals with high-temperature acclimation history produced more beneficial substances for metabolic homeostasis. The findings revealed the critical role of mitochondria in heat response of animals and provided insight into the heat adaptation of marine molluscs.

## Supplementary information

Supplementary Information.

Supplementary Table S1.

Supplementary Table S2.

## Data Availability

All raw data files are available on the NIH Metabolomics Workbench website (DataTrack ID: 1913). The datasets used and analyzed during the current study are also available from the corresponding author on reasonable request.
