# Supplementary material for: Metabolomics Adaptation of Juvenile Pacific Abalone Haliotis discus hannai to Heat Stress
Source: Sci Rep. 2020 Apr 14;10:6353. doi: 10.1038/s41598-020-63122-4 (PMC7156721; doi:10.1038/s41598-020-63122-4)
Supplement: Supplementary file 1 — Supplementary Information. [file 41598_2020_63122_MOESM1_ESM.pdf]

Supplementary information for

Metabolomics Adaptation of Juvenile Pacific Abalone *Haliotis discus hannai* to Heat Stress

Fei Xu[1*], Tingting Gao[1,3], Xiao Liu[1,2]

[1] Key Laboratory of Experimental Marine Biology, Center for Mega-Science, Institute of Oceanology, Chinese Academy of Sciences, Qingdao, 266071, China
[2] School of Fishery, Zhejiang Ocean University, Zhoushan, 316022, China
[3] University of Chinese Academy of Sciences, Beijing, 10049, China
*corresponding author: xufei@qdio.ac.cn

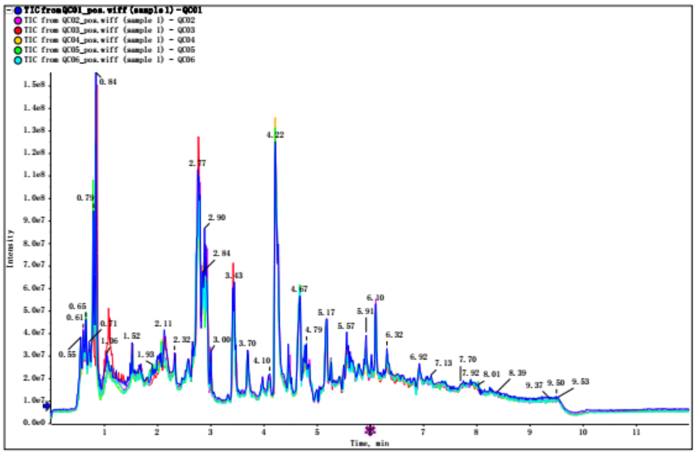

Supplementary Figure S1 Total ion chromatogram of quality control (QC) samples in positive ion model detected by UHPLC-QTOF-MS. Image was created by software Analyst TF 1.7 (https://sciex.com/products/software/analyst-tf-software) coupled with TripleTOF® 6600 System (AB Sciex, USA).

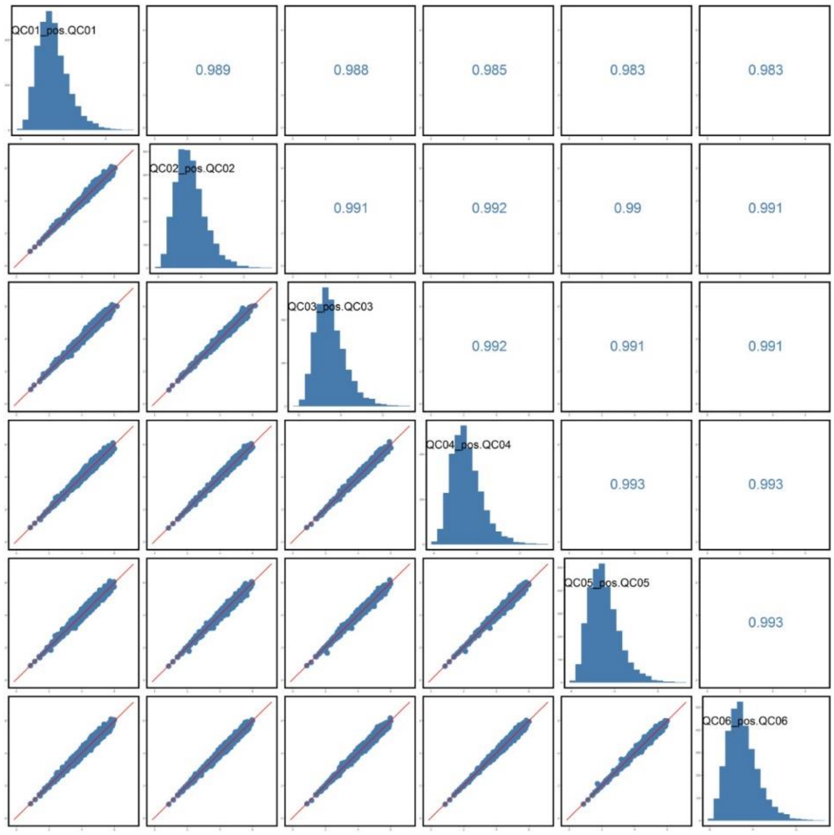

Supplementary Figure S2 Correlation analysis of quality control (QC) samples in positive ion model. Image was created by R software version 3.5.3 (https://www.r-project.org/).

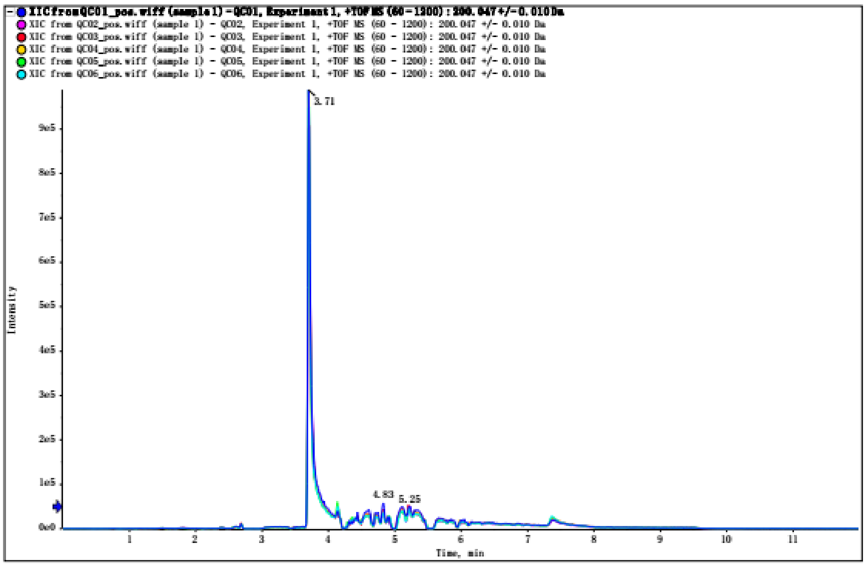

Supplementary Figure S3 Extracted ion chromatogram of interior label (L-chloro-2-phenylalanine) in quality control (QC) samples under positive ion model. Image was created by software Analyst TF 1.7 (https://sciex.com/products/software/analyst-tf-software) coupled with TripleTOF® 6600 System (AB Sciex, USA).

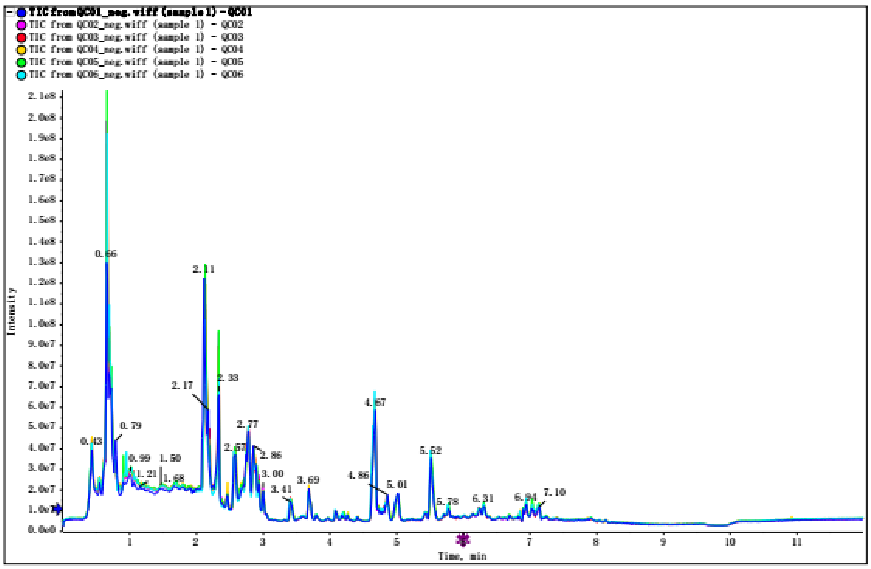

Supplementary Figure S4 Total ion chromatogram of quality control (QC) samples under negtive ion model detected by UHPLC-QTOF-MS. Image was created by software Analyst TF 1.7 (https://sciex.com/products/software/analyst-tf-software) coupled with TripleTOF® 6600 System (AB Sciex, USA).

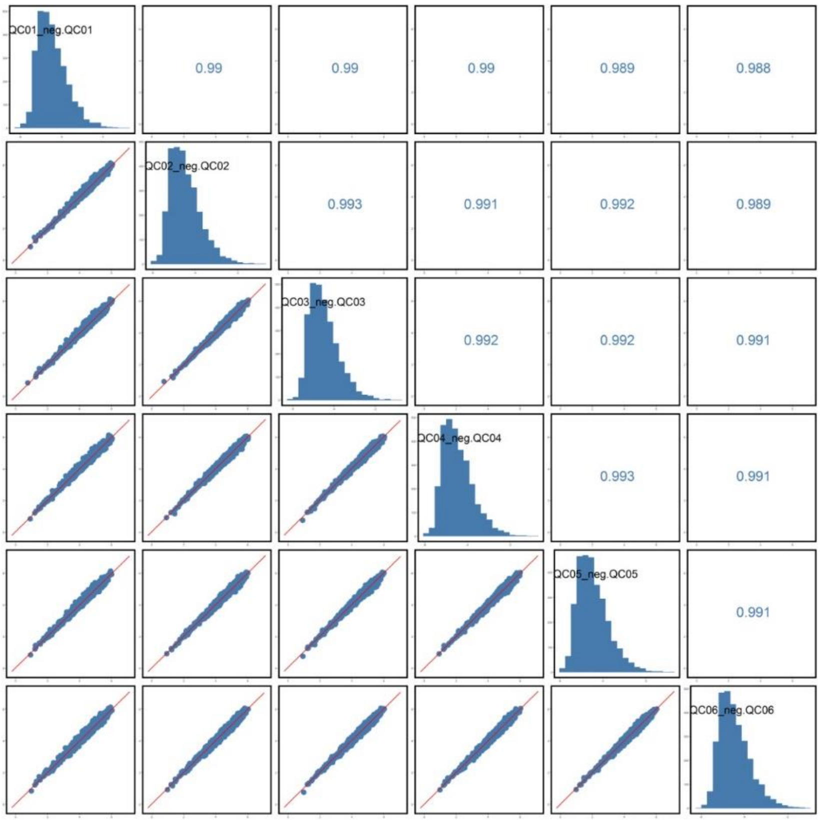

Supplementary Figure S5 Correlation analysis of quality control (QC) samples under negtive ion model. Image was created by R software version 3.5.3 (https://www.r-project.org/).

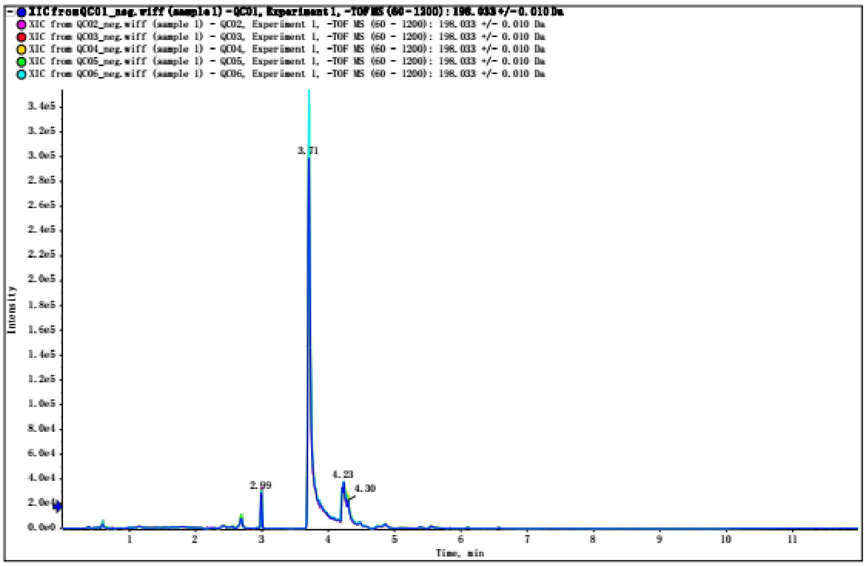

Supplementary Figure S6 Extracted ion chromatogram of interior label (L-chloro-2-phenylalanine) in quality control (QC) samples under positive ion model. Image was created by software Analyst TF 1.7 (https://sciex.com/products/software/analyst-tf-software) coupled with TripleTOF® 6600 System (AB Sciex, USA).

Supplementary Table S1 Annotated differential metabolites of L-LH pair.

See excel file.

Supplementary Table S2 Annotated differential metabolites of H-HH pair.

See excel file.

Supplementary Table S3 Metabolite biomarkers of L-LH pair.

Name	FC	*P*	VIP	Regulated
**Nicotinamide**	11.53	5.63E-05	2.08	up
**2-Methylbutyroylcarnitine**	9.55	3.90E-06	2.36	up
**(-)-Naringenin**	8.74	3.51E-03	1.68	up
**Maleamic acid**	5.94	4.34E-05	2.08	up
Cytosine	5.71	8.37E-04	1.84	up
**O-Acetyl-L-serine**	5.16	7.84E-07	2.20	up
**Stearoylcarnitine**	4.15	1.00E-03	1.97	up
**Iminodiacetic acid**	3.6	5.56E-06	2.34	up
**1,3-Dimethyluric acid**	3.54	1.20E-06	2.21	up
L-Gulonic gamma-lactone	2.83	4.71E-04	1.85	up
**CDP-choline**	2.72	8.79E-06	2.26	up
Phenoxybenzamine	2.55	7.38E-03	1.68	up
**D-Biotin**	2.24	6.77E-04	2.05	up
**1,7-Dimethyluric acid**	2.19	2.78E-03	1.78	up
**Acamprosate**	2.11	1.90E-04	1.85	up
**Amrinone**	2.08	3.43E-06	2.04	up
**Erythrono-1,4-lactone**	2.07	2.18E-08	2.42	up
**Niflumic Acid**	2.06	1.03E-03	1.85	up
Lavandulol	2.06	5.57E-04	2.00	up
**5-Hydroxyindoleacetate**	1.98	1.07E-03	1.75	up
**MDMA**	1.96	3.17E-07	2.16	up
**alpha-Guanidinoglutaric Acid**	1.95	4.93E-06	2.28	up
**DL-Indole-3-lactic acid**	1.93	8.58E-06	2.14	up
**N-Acetyl-L-tyrosine**	1.92	3.03E-03	1.72	up
**5-Methylcytidine**	1.86	6.25E-05	2.21	up
**Suberic acid**	1.85	2.45E-03	1.76	up
**L-Kynurenine**	1.81	5.08E-05	2.18	up
**L-Palmitoylcarnitine**	1.78	1.35E-02	1.54	up
**Creatine**	1.75	3.03E-05	2.08	up
beta-Alanine	1.74	5.48E-05	2.09	up
**Phenelzine**	1.73	1.15E-05	2.14	up
Glutaric acid	1.62	1.66E-03	1.89	up

**7-Methylxanthine**	1.54	9.78E-04	1.69	up
**Imidazoleacetic acid**	1.52	2.90E-03	1.70	up
Leu-Thr	0.61	5.44E-03	1.69	down
Diethylcarbamazine	0.6	1.38E-04	1.96	down
Compactin	0.59	2.05E-03	1.77	down
DL-alpha-Phenylglycine	0.58	1.14E-03	1.90	down
Fexofenadine	0.57	3.17E-03	1.58	down
1-Oleoyl-sn-glycero-3-phosphocholine	0.53	6.31E-04	1.97	down
1-Salicylate glucuronide	0.48	3.59E-03	1.79	down
Meclofenoxate	0.46	2.54E-03	1.79	down

Note： 'A-B' was used to name sets of DEMs, such as L-LH and H-HH. Up-regulated metabolite contents in the sample B were higher than the sample A; down-regulated metabolites were opposite. Bold words indicate overlapped metabolite biomarkers between L-LH and H-HH.

Supplementary Table S4 Metabolite biomarkers of H-HH pair.

**Name**	**FC**	***P***	**VIP**	**Regulated**
**Nicotinamide**	11.21	1.88E-02	1.55	up
**(-)-Naringenin**	6.22	5.67E-03	1.77	up
**O-Acetyl-L-serine**	5.47	4.88E-03	1.78	up
Pro-Tyr	5.14	9.23E-04	2.15	up
**Maleamic acid**	5.06	5.89E-03	1.74	up
**2-Methylbutyroylcarnitine**	4.55	2.80E-04	2.25	up
**Stearoylcarnitine**	3.71	2.34E-05	2.46	up
**1,3-Dimethyluric acid**	3.59	4.22E-05	2.26	up
**Iminodiacetic acid**	3.45	1.10E-04	2.38	up
**1,7-Dimethyluric acid**	3.33	2.21E-02	1.6	up
D-Glucose 6-phosphate	3.2	1.37E-02	1.68	up
**CDP-choline**	3.11	1.40E-03	2.12	up
**Amrinone**	2.8	3.99E-04	2.06	up
**DL-Indole-3-lactic acid**	2.67	6.72E-05	2.36	up
**Suberic acid**	2.34	1.09E-03	2.14	up
Ammelide	2.33	7.10E-05	2.17	up
**alpha-Guanidinoglutaric Acid**	2.33	3.42E-05	2.36	up
**Creatine**	2.19	2.33E-05	2.37	up
**D-Biotin**	2.19	1.20E-02	1.75	up
Pro-Asn	2.13	5.52E-04	2.15	up
**MDMA**	2.11	6.18E-05	2.24	up
Thr-Ala	2.1	4.08E-04	2.09	up
**Phenelzine**	2.08	1.72E-05	2.35	up
S-Methyl-5'-thioadenosine	2.07	6.65E-03	1.83	up
4-Hydroxycinnamic acid	2.05	2.54E-05	2.31	up
**7-Methylxanthine**	2.05	3.43E-04	1.95	up
**N-Acetyl-L-tyrosine**	2.04	1.78E-02	1.59	up
**Acamprosate**	2.03	1.39E-02	1.6	up
trans-2-Hydroxycinnamic acid	2.02	5.25E-06	2.39	up
Theobromine	2.01	6.87E-03	1.7	up
N-Acetyl-D-glucosamine	2	1.91E-03	1.9	up
Tiopronin	1.99	5.42E-05	2.23	up

**Name**	**FC**	***P***	**VIP**	**Regulated**
L-Phenylalanine	1.97	1.10E-02	1.66	up
Dimethylglycine	1.95	6.11E-03	1.78	up
Phe-Ala	1.92	1.74E-02	1.52	up
Ajmalicine	1.87	4.52E-03	1.8	up
Benzoylecgonine	1.87	6.65E-03	1.81	up
beta-Octylglucoside	1.86	1.37E-02	1.61	up
Trimethobenzamide	1.86	5.41E-03	1.71	up
Ser-Ala	1.86	7.34E-04	1.97	up
Glucosamine	1.85	5.22E-04	1.98	up
Lys-Ile	1.84	4.93E-03	1.79	up
N-Methyl-D-aspartic acid	1.82	2.06E-03	1.84	up
Thr-Thr	1.81	9.68E-04	1.93	up
Pro-Gly	1.8	1.17E-03	1.93	up
Taurolithocholic acid	1.79	2.02E-03	1.9	up
**Erythrono-1,4-lactone**	1.79	2.22E-04	2.29	up
1-Palmitoyllysophosphatidylcholine	1.78	1.33E-02	1.61	up
Gly-Val	1.75	6.94E-03	1.68	up
Linalool oxide	1.75	5.50E-04	2	up
trans-2-Octenoic acid, ethyl ester	1.74	1.65E-02	1.54	up
**5-Methylcytidine**	1.72	3.89E-03	1.95	up
**5-Hydroxyindoleacetate**	1.72	1.09E-03	1.84	up
Trp-Cys	1.71	1.22E-03	2	up
Val-Ala	1.71	4.27E-03	1.74	up
ADP-ribose	1.7	1.34E-02	1.56	up
**Niflumic Acid**	1.7	1.40E-02	1.65	up
IBMX	1.69	1.60E-02	1.64	up
4-Hexen-1-ol, (E)-	1.69	9.77E-04	1.94	up
Dihydrouracil	1.68	2.91E-03	1.74	up
Ketanserin	1.68	5.99E-03	1.64	up
Phenethyl Caffeiate	1.68	6.39E-03	1.75	up
L-Alanine	1.67	7.65E-03	1.67	up
Pro-Val	1.67	3.27E-03	1.77	up
Hypotaurine	1.65	2.98E-02	1.5	up
3-Methylphenylacetic acid	1.64	1.19E-02	1.52	up

Name	FC	*P*	VIP	Regulated
Quinate	1.64	7.87E-03	1.58	up
**L-Kynurenine**	1.64	1.34E-02	1.73	up
Tyramine	1.64	7.53E-03	1.69	up
Coumarin	1.63	1.07E-02	1.62	up
Ibuprofen	1.63	4.18E-05	2.35	up
isocarboxazid	1.63	1.58E-02	1.52	up
Procaine	1.62	1.67E-02	1.6	up
**L-Palmitoylcarnitine**	1.62	4.65E-03	1.8	up
Thr-Ser	1.62	9.00E-03	1.64	up
Tyr-Leu	1.6	1.46E-02	1.59	up
(R)-mevalonic acid 5-Phosphate	1.6	1.34E-02	1.54	up
Ile-Ala	1.6	9.88E-03	1.6	up
**Imidazoleacetic acid**	1.58	2.93E-03	1.96	up
Triflupromazine	1.58	1.23E-02	1.58	up
DL-Homoserine	1.57	9.97E-03	1.59	up
Met-Ala	1.56	2.00E-03	1.86	up
AG-17	1.54	5.34E-03	1.75	up
Verapamil	1.54	2.26E-02	1.53	up
Phenyllactic acid	1.53	1.84E-02	1.56	up
Pyrrolidine	1.53	5.15E-06	2.39	up
Sinapyl alcohol	1.53	2.30E-02	1.52	up
Val-Tyr	1.52	9.54E-03	1.63	up
L-Arabitol	1.52	8.65E-03	1.61	up
Desoxypeganine	1.51	1.62E-02	1.51	up
L-Histidine	1.51	4.50E-03	1.75	up
DL-Methionine sulfoxide	1.5	1.07E-03	2.05	up
3-Methyluridine	0.59	2.24E-03	1.83	down
N- alpha -Acetyl-L-arginine	0.53	8.40E-04	2.14	down
D-Mannose 1-phosphate	0.51	1.19E-02	1.73	down
3.alpha.-Mannobiose	0.47	1.71E-02	1.59	down
Nomilin	0.46	2.04E-04	2.15	down
D(-)-beta-hydroxy butyric acid	0.45	8.91E-03	1.62	down
Salidroside	0.44	1.67E-02	1.52	down
Cytidine 2',3'-cyclic phosphate	0.42	5.94E-05	2.22	down

Note： 'A-B' was used to name sets of DEMs, such as L-LH and H-HH. Up-regulated metabolite contents in the sample B were higher than the sample A; down-regulated metabolites were opposite. Bold words indicate overlapped metabolite biomarkers between L-LH and H-HH.
